# Clinical Efficacy and Quality of Life Follow-Up of Reconstructive Endovascular Therapy for Acute Intracranial Vertebral Artery Dissection Aneurysms

**DOI:** 10.3389/fsurg.2020.00032

**Published:** 2020-07-29

**Authors:** Lu Gao, Yu Qian, Jing Luo, Yang Hong, Yangchun Hu, Hongwei Cheng, Baochun Cheng

**Affiliations:** Department of Neurosurgery, First Affiliated Hospital of Anhui Medical University, Heifei, China

**Keywords:** vertebral artery dissection aneurysms, reconstructive endovascular therapy, quality of life, functional outcome, cognitive status

## Abstract

**Background:** Intracranial vertebral artery dissection aneurysms (VADAs) may cause acute ischemia or hemorrhage, in which case urgent endovascular treatment will be needed. Although the majority of patients obtain a good functional outcome after surgery, a surprising finding has been a poor quality of life (QOL) in follow-up. The purpose of this study was to evaluate clinical efficacy in reconstructive endovascular therapy for acute intracranial VADAs and to analyze the factors contributing to subsequent QOL.

**Methods:** In this prospective study, 33 consecutive VADA patients with subarachnoid hemorrhage were recruited for comparison with 37 VADA patients with posterior circulation cerebral ischemia. All VADA patients were treated using a reconstructive strategy. Clinical, radiological, neurological, and cognitive data, as well as QOL, were assessed at admission and 6 months after surgery. Stoke Specific Quality of Life (SS-QOL) was evaluated for patients with good functional outcome [modified Ranking Scale (mRS) scoring 0-2] for subgroup analysis. Predictors for QOL at follow-up were analyzed by regression model.

**Results:** Immediate angiography after surgery showed complete VADA obliteration in 57 (81.4%) patients and partial obliteration in 13 (18.6%) patients. Three (4.3%) cases suffered from perioperative complications, comprising two cases of stent thrombosis in the hemorrhagic group and one case of posterior inferior cerebellar artery occlusion in the ischemic group. Twenty-five (75.8%) patients in the hemorrhagic group and 30 (81.1%) patients in the ischemic group had a favorable outcome (mRS scoring 0-2) at 6-month follow-up. Follow-up angiography displayed that one case of recurrence occurred separately in both groups. Fifteen of the 33 hemorrhagic patients (45.5%) and 19 of the 37 ischemic patients (51.4%) rated QOL at follow-up as bad (SS-QOL score ≤ 3.9) despite a good functional outcome. Severity of neurological disorder and impaired neurocognition at baseline in VADA patients are proved to be independent predictors for the decline of QOL according to regression analysis.

**Conclusion:** Reconstructive endovascular therapy for acute intracranial VADAs is a safe and effective method with a low complication rate. VADAs lead to impaired QOL at 6-month follow-up, which is attributable to multiple factors. This study demonstrated that neurological and cognitive status at baseline is of significant importance for QOL after VADAs.

## Introduction

Intracranial vertebral artery dissection aneurysms (VADAs) are increasingly diagnosed where there is presentation with subarachnoid hemorrhage and posterior circulation ischemia (1, 2). In the literature, the annual incidence of spontaneous VADAs has been estimated at 1–1.5/100,000 for all age groups (2, 3). Intracranial VADAs can result in an acute cerebrovascular event, especially in middle-aged patients, and are associated with high mortality and morbidity due to the risk of recurrent bleeding (4, 5). Therefore, early treatment is necessary for symptomatic and progressive VADA because of its tendency to progress and the high risk of fatal bleeding.

Endovascular therapy, including deconstructive and reconstructive methods, is favored for the treatment of intracranial VADAs (6). Although most intracranial VADAs can be treated with a deconstructive approach, a cerebrovascular ischemic event and re-bleeding still happen in some patients (7). Furthermore, the application of the deconstructive approach by parent artery sacrifice is limited when the hypoplastic contralateral vertebral artery cannot provide adequate posterior circulation blood supply following an ipsilateral vertebral artery occlusion (8). With the increasing development of the capabilities of stents and embolization materials, an endovascular reconstructive method with or without stents is preferred for the treatment of acute intracranial VADAs (9).

It is well-known that previous study of intracranial VADAs mainly focused on clinical effects, such as preoperative complications, mortality, and recurrence rate. However, in recent years, the quality of life of those patients receiving reconstructive endovascular treatment has become more and more important. Quality of life after vertebral artery dissection has been examined in a standardized manner in only three published articles in the last few decades (10–12). Also, a subarachnoid hemorrhage study explored the relationship of quality of life and clinical outcomes (13). These studies consistently showed that the quality of life was impaired despite relatively good clinical outcomes. Nevertheless, there is still a lack of research exploring quality of life after endovascular reconstructive therapy for intracranial VADAs.

The purpose of this study was therefore as follows: (1) to evaluate clinical outcomes, such as the radiological features, preoperative complications, and recurrence rate, of using reconstructive endovascular therapy for intracranial VADAs. (2) To explore the clinical, neurological, and cognitive status characteristics of intracranial VADA patients before and after surgery. (3) To identify the predictive factors for quality of life at 6-month follow-up in reconstructive therapy patients.

## Methods

### Study Design and Participants

From January 2017 to September 2019, all patients admitted to our Neurosurgery department diagnosed with intracranial VADAs due to acute cerebral stroke were enrolled in our study. Finally, a total of 33 cases with subarachnoid hemorrhage (group H) and 37 cases with posterior circulation ischemia (group I) of the intracranial VADA patients satisfied our study requirements. All data were prospectively collected before and 6 months following surgery.

The acute subarachnoid hemorrhage in group H patients was confirmed by computed tomography (CT), and the group I patients were diagnosed on the basis of acute cerebral infarction in a posterior blood supply area being seen in a magnetic resonance image (MRI) or transient ischemic attack (TIA) of the posterior circulation. The diagnosis of all intracranial VADAs was based on cerebral digital subtraction angiography (DSA). Endovascular reconstructive therapy was performed for these patients. All subjects met the following inclusion criteria: (1) a reliable intracranial VADA diagnosis; (2) undergoing endovascular reconstructive therapy; (3) a stable psychological and physical condition for examination, i.e., no consciousness disorder, and, for subarachnoid hemorrhage patients, Hunt-Hess grades I-II for language competence fluency.

Exclusion criteria were: (1) intracranial VADA being due to severe trauma; (2) subarachnoid hemorrhage or cerebral ischemia because of anterior circulation aneurysms; (3) preexisting or concurrent central nervous system diseases or psychological disorders; (4) alcohol abuse or medication usage that may lead to cognitive damage.

Participants conforming to our study criteria were examined for psychological, cognitive, and neurological factors. The data collection in each patient was performed at baseline in the acute phase in the hospital and at 6-month follow-up after surgery. The following items were recorded: clinical data, radiological character, cognitive testing, and mRS scoring, as well as quality of life.

The study protocol was approved by the Ethics Committee of Anhui Medical University. All participants gave written informed consent.

### Clinical and Neurological Assessment

Clinical data collection comprised a history of hypertension, diabetes mellitus, and dyslipidemia, as well as consciousness status and clinical presentation as evaluated at baseline. Neurological status was evaluated by an experienced neurologist using the modified Rankin Scale (mRS) (14). The mRS is considered worldwide to be the most reliable functional outcome measure after stroke. Consistent with the previous interpretations, an mRS score of 0-2 reflects a good functional outcome, meaning an independent daily living competence.

### Radiological Examination

Patients with suspected intracranial VADAs received CT and DSA examinations. If a VADA patient was considered to have posterior circulation ischemia, an additional MRI examination was performed for them. DSA confirmed the VADA diagnosis and showed the size, shape, and location of intracranial VADAs. In our study, the main angio-architecture was divided into two groups: the dilatation without stenosis group and the dilatation with stenosis group. For group H patients, the severity of subarachnoid hemorrhage was defined according to the CT Fisher grade. Furthermore, the extension of posterior circulation area acute infarction in cases of group I was measured by MRI and categorized into either a maximal diameter >10 or ≤10 mm. All patients received DSA examination at 6-month follow-up.

### QOL Measurement

The quality of life in the week before admission to hospital and at follow-up was evaluated by the German Version of the Stroke-Specific Quality Of Life Scale (SS-QOL) (15, 16). This generates 12 domain-related scores and a total score. A total score ≥ 4.0 was considered a good QOL and a score ≤3.9 a poor QOL, according to the Fisher et al. study (11), which presumed that the mean SS-QOL in patients before dissection is the best measure for good QOL.

### Neuropsychological Assessment

The neuropsychological status was assessed at baseline and the follow-up using the Mini-Mental State Examination (MMSE) (17) and the more sensitive Montreal Cognitive Assessment (MoCA) (18) as cognitive screening tools.

### Endovascular Therapy

All procedures were performed under general anesthesia. DSA was also used to assess the relation between the aneurysm and the posterior inferior cerebellar artery (PICA). Group I patients were given a dose of double antiplatelets (100 mg aspirin + 75 mg clopidogrel, one time per day) 3 days before surgery. Group H patients were treated immediately after admission due to concern regarding rebleeding. If constructive endovascular management with a stent was planned, patients received intravenous heparin at the beginning of the procedure with an activated clotting time of between 250 and 300 s during the procedure. After surgery, patients that had undergone treatment with a stent were administered heparin for 3 days (40 mg, every 12 h, hypodermic injection). Then, these patients were kept on a dual antiplatelet treatment for 6 weeks, with 100 mg aspirin+75 mg clopidogrel, followed by aspirin (100 mg) monotherapy indefinitely. Patients treated without a stent had to be evaluated by a neurologist after surgery to determine whether they needed to take antiplatelet drugs. Angiographic follow-up was performed with DSA at 6 months after surgery. The immediate and follow-up obliteration grade was defined as complete or partial obliteration according to a previous study (19).

### Statistical Analysis

The statistical analysis was performed using SPSS Statistics Version 19.0. Demographic and clinical data were examined through descriptive analysis, calculating mean values with standard deviations for metric variables, and frequency calculation for categorical variables. The data at baseline and follow-up were compared using the Mann-Whitney U test for continuous variables, and the X^2^ test and its continuity correction or Fisher's exact test was used for categorical variables, as appropriate. Subgroup analyses were performed for patients with mRS 0–2 and SS-QOL≥4.0 vs. those with mRS 0–2 and SS-QOL ≤3.9. Then, univariate model linear regression was used to test the predictive power of certain variables for the variance of SS-QOL scores at follow-up. Afterward, a multivariate regression model was sued to assess the variables that had proven statistically significant in the univariate analysis. *P* < 0.05 was considered statistically significant.

## Results

### Clinical Baseline Data

Thirty-three patients with subarachnoid hemorrhage and 37 patients with posterior circulation ischemia fulfilled our study inclusion criteria and were included in the study as group H and group I, respectively. The demographic and clinical data of the two groups are shown in Table 1. All patients presented with a sudden onset of intense headache with or without nausea and vomiting in the group H. The Hunt-Hess grade in group H patients was I-II, without consciousness disorder. Twenty-eight (75.7%) patients presented with dizziness or vertigo as the main symptom in group I. The remaining patients experienced transient ischemic attack or acute posterior circulation infarction.

**Table 1:** Demographic and clinical characteristics at admission.

	**Group H (*n* = 33)**	**Group I (*n* = 37)**
Age	51.6 ± 6.9	52.8 ± 7.8
Gender		
Male n (%)	21 (63.6%)	15 (40.5%)
Female n (%)	12 (36.4%)	22 (59.5%)
Education, n (%)		
Above high school	8 (24.2%)	13 (35.1%)
Below high school	25 (75.8%)	24 (64.9%)
Employment n (%)		
Yes	24 (72.7%)	28 (75.7%)
No	9 (27.3%)	9 (24.3%)
Living arrangement n (%)		
Alone	3 (9.1%)	4 (10.8%)
With family	30 (90.9%)	33 (89.2%)
Cerebral ischemia history n (%)		
Infarction lesion	3 (9.1%)	6 (16.2%)
TIA	6 (18.2%)	7 (18.9%)
Neurovascular risk factors n (%)		
Hypertension	11 (33.3%)	14 (37.8%)
Diabetes mellitus	8 (24.2%)	10 (27.0%)
Hypercholesterolemia	7 (21.2%)	11 (29.7%)
Smoking	13 (39.4%)	12 (32.4%)

*Group H patients had subarachnoid hemorrhage, group I patients had ischemia*.

### Functional, Cognitive Status, and QOL

The recruited patients were tested by experienced neurologists, who were blind to this study, for functional, cognitive status and QOL at admission. The mRS scoring at baseline of the two groups was low compared with normal people, indicating that the affected patients had impaired neurological status. The two cognitive screening tests (MMSE and MoCA) also demonstrated the impairment of cognitive function in both groups. Likewise, there was a decline of the mean value of the total quality of life score as measured by SS-QOL (Table 3).

### Radiological Data and Endovascular Therapy Outcome

**Figure 1:**
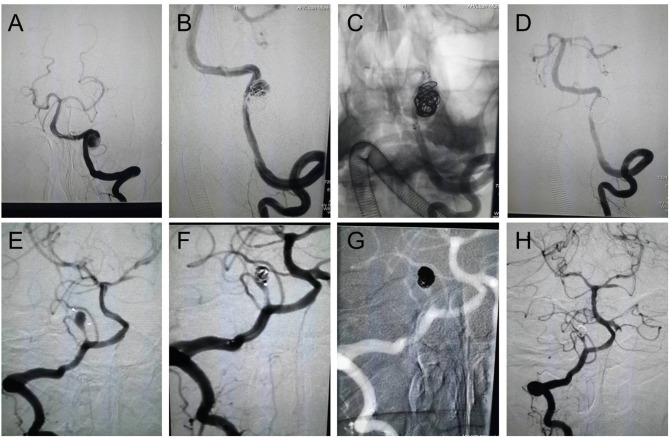
Endovascular reconstructive treatment of intracranial VADAs. **(A–D)** A ruptured VADA was treated with a single stent and coils, showing complete obliteration; **(E–H)** An unruptured PICA area VADA was treated with coils, showing partial obliteration.

According to CT scan and Fisher grade evaluation in group H, we found four Fisher grade I patients, 12 grade II patients, 11 grade III patients, and six grade IV patients. In group I, we observed that the most frequent cerebral infarction locations were the cerebellum and the brainstem. The MRI showed that the infarction size of six patients was larger than 10 mm and that the infarction size was smaller than 10 mm in 10 patients. Finally, the DSA identified the location, size, and form of intracerebral VADAs in both groups (Table 2). All ruptured aneurysms were reconstructed by using coils and stent(s) (single stent, *n* = 24; double stents, *n* = 9), achieving a 100% technical success rate. Two cases of stent-related intraoperative thrombosis occurred; these were treated by using tirofiban and a favorable outcome was achieved. The group I patients were treated using coils with or without stent(s) (no stent, *n* = 9; single stent, *n* = 20; double stents, *n* = 8), and all achieved technical success as well. Only one patient encountered a treatment-related complication, namely posterior inferior cerebellar artery occlusion; however, this patient recovered very well. The rate of immediate obliteration grade for both groups was 81.4% (*n* = 57). Most of the partial obliterated aneurysms were found in the cases involving PICA (*n* = 7, 53.8%) (Figure 1, Table 2).

**Table 2:** Radiological and endovascular treatment outcome.

	**Group H (*n* = 33)**	**Group I (*n* = 37)**
SAH Fisher grade (n%)		
I	4 (12.1%)	
II	12 (36.4%)	
III	11 (33.3%)	
IV	6 (18.2%)	
Infarction size (n%)		
>10 mm		6 (16.2%)
≤ 10 mm		10 (27.0%)
Aneurysm dimension (n%)		
>10 mm	23 (69.7%)	22 (59.5%)
≤ 10 mm	10 (30.3%)	15 (40.5%)
Aneurysm type (n%)		
Dilatation without stenosis	17 (51.5%)	20 (54.1%)
Dilatation with stenosis	15(45.5%)	17 (45.9%)
PICA involvement (n%)		
Yes	6 (18.2%)	9 (24.3%)
No	27 (81.8%)	28 (75.7%)
Operation time (n%)		
>3 h	9 (27.3%)	11 (29.7%)
≤ 3 h	24 (72.7%)	26 (70.3%)

*SAH, subarachnoid hemorrhage; PICA, posterior inferior cerebral artery*.

### Clinical Follow-Up

Clinical follow-up of all patients was available at 6 months. In angiographic follow-up, we observed that one case of aneurysm recurrence occurred separately in both groups. Twenty-five (75.8%) patients in the hemorrhagic group and 30 (81.1%) patients in the ischemic group had a favorable outcome (mRS scoring 0–2) at 6-month follow-up. However, 15 patients (45.5%) in group H and 19 patients (51.4%) in group I rated QOL as bad (SS-QOL score ≤ 3.9) despite the good functional outcome in each group. The mean values of mRS, MMSE, and MCoA scoring improved from the baseline to follow-up, but this was statistically significant only in the mRS (*p* < 0.05). We analyzed the change of mean total scores of QOL, as measured by SS-QOL, from baseline to follow-up and found a significant deterioration (*p* < 0.05) (Table 3). With further analysis of changes in scores in the twelve SS-QOL domains from baseline to follow-up in the two groups, we discovered that impairments were mainly in psychosocial domains and less common in physical domains.

**Table 3:** Functional, cognitive and QOL outcome at baseline and follow-up.

	**Group H (*****n*** **=** **33)**		**X^**2**^,F**	**p**	**Group H (*****n*** **=** **33)**		**X^**2**^,F**	**p**
	**Baseline**	**Follow-up**	**H**		**Baseline**	**Follow-up**	**H**	
SS-QOL	4.38 ± 0.42	3.77 ± 0.62	8.78	0.012	4.62 ± 0.34	4.17 ± 0.59	7.65	0.034
SS-QOL≥4.0		10 (30.3%)				11 (29.7%)		
SS-QOL ≤ 3.9		23 (69.7%)				26 (70.3%)		
mRS	2.42 ± 1.31	1.63 ± 1.22	10.60	0.004	2.09 ± 1.38	1.39 ± 1.12	6.36	0.036
mRS 0-2		25 (75.8%)				30 (81.1%)		
mRS 3-5		8 (24.2%)				7 (18.9%)		
MMSE	27.47 ± 1.82	27.92 ± 1.30	3.69	0.157	28.21 ± 1.36	28.98 ± 1.04	3.63	0.162
MoCA	24.23 ± 3.67	25.22 ± 2.92	1.52	0.223	25.17 ± 3.46	25.86 ± 3.28	2.31	0.314

*Statistical significance by Mann-Whitney and Fisher's exact tests, as appropriate*.

*SS-QOL, stroke-specific quality of life; MMSE, mini-mental state examination; MoCA, Montreal cognitive assessment*.

### Subgroup Analysis of Poor QOL in Good Functional Outcome

SS-QOL scores at follow-up were stratified for good functional outcome (mRS 0-2) plus good quality of life (SS-QOL≥4.0) vs. good functional outcome (mRS 0-2) plus bad quality of life (SS-QOL ≤ 3.9). Fifteen of the group H patients (45.5%) showed a bad quality of life despite a good functional outcome, and ten of the patients (30.3%) had a good quality of life combined with a good functional outcome. In group I, nineteen patients (51.4%) displayed a bad quality of life and a good functional outcome, whereas eleven (29.7%) had a good quality of life plus a good functional outcome. Further subgroup analysis was performed in the two groups to identify potential affecting variables such as age, gender, radiological feature, aneurysm type, operation time, or cognitive status at baseline. The main findings were in group H patients with bad QOL, who had significantly higher Fisher grade and lower mean values for MMSE and MoCA at baseline. Likewise, group I patients with bad QOL also had significantly lower mean values of MMSE and MoCA at baseline (Table 4).

**Table 4:** Subgroup analysis of patients with mRS 0-2 at follow-up.

**Variable**	**Group H**		**X^**2**^,F**	**p**	**Group H**		**X^**2**^,F**	**p**
	**SS-QOL**		**U**		**SS-QOL**		**U**	
	**≥ 4.0**	**≤ 3.9**			**≥ 4.0**	**≤ 3.9**		
	***n* = 10**	***n* = 15**			***n* = 11**	***n* = 19**		
Age	50.3 ± 5.4	50.8 ± 6.3	80.5	n.s.	51.2 ± 6.1	52.7 ± 6.7	78.3	n.s.
Gender (male, n%)	6 (60.0%)	8 (53.3%)	68	n.s.	5 (45.5%)	10 (52.6%)	65	n.s.
**Fisher grade (n%)**								
I–II	6 (60.0%)	5 (33.3%)						
III–IV	6 (60.0%)	10 (66.7%)	42	0.038				
**Infarction size (n%)**								
>10 mm					2 (18.2%)	3 (15.9%)		
≤ 10 m					2 (18.2%)	4 (21.1%)	92.5	n.s.
**Operation time (n%)**								
>3 h	3 (30.0%)	4 (26.7%)			2 (18.2%)	4 (21.1%)		
≤ 3 h	7 (70.0%)	11 (73.3%)			9 (81.8%)	15 (78.9%)	88.3	n.s.
MMSE at baseline	28.47 ± 1.08	26.92 ± 1.34	46	0.042	28.78 ± 0.92	27.04 ± 1.38	42	0.039
MoCA at baseline	25.86 ± 1.12	24.27 ± 1.45	43	0.040	26.06 ± 1.06	24.84 ± 1.42	40	0.032

*Statistical significance by Mann-Whitney and Fisher's exact tests, as appropriate*.

*SS-QOL, stroke-specific quality of life; MMSE, mini-mental state examination; MoCA, Montreal cognitive assessment; n.s. not significant*.

### Predictors of QOL

The preceding analysis of this study indicated that quality of life may be correlated with neurocognitive measures, such as MMSE and MoCA at baseline, and with neurological status, such as mRS at baseline. Therefore, regression analysis was performed to explore the potential predictors of quality of life. Univariate linear regression analysis showed that cognition scores (MMSE, MoCA) at baseline, mRS scores at baseline, and Fisher grade in group H, and the MMSE, MoCA, and mRS scores at baseline in group I were predictors of quality of life at follow-up. In the subsequent multiple regression analysis, MMSE and mRS scores at baseline in both groups were proved to be the independent factors for quality of life at follow-up. Thus, reduced neurocognition and neurological function status at baseline were predictive for a poor quality of life at follow-up in VADA patients who had undergone endovascular reconstruction therapy (Table 5).

**Table 5:** Regression analysis of predictors for SS-QOL at follow-up in VADA patients.

	**Group H**		**Group I**	
	***R*^**2**^**	***p***	***R*^**2**^**	***p***
**Univariate linear regression analysis**				
**Independent variable**				
Age	0.070	n.s.	0.067	n.s.
Gender	0.002	n.s.	0.001	n.s.
Fisher grade	0.263	0.03		
Infarction size			0.198	n.s.
Operation time	0.056	n.s.	0.072	n.s.
mRS at baseline	0.180	0.019	0.210	0.016
MMSE at baseline	0.385	0.036	0.463	0.028
MoCA at baseline	0.212	0.024	0.312	0.016
**Multivariate linear regression analysis**				
mRS at baseline	0.128	0.003	0.168 0.027	
MMSE at baseline	0.274	0.004	0.352 0.003	

*SS-QOL, stroke-specific quality of life; MMSE, mini-mental state examination; MoCA, Montreal cognitive assessment; n.s., not significant*.

## Discussion

This investigation is, to the best of our knowledge, the first study to evaluate the factors influencing quality of life at 6-month follow-up after endovascular reconstructive therapy for intracranial VADAs using a prospective and comparative study design. We found the endovascular reconstructive treatment is an effective and safe strategy, with a low incidence rate of complications, for intracranial VADAs in the acute phase. Acute intracranial VADAs can lead to impairment of neurocognitive status (MMSE, MoCA), neurological functional outcome (mRS) at baseline, and quality of life at follow-up. Simultaneously, we discovered that the neurological functional outcome (mRS score) was improved significantly in VADA patients at 6-month follow-up, but most of them had a poor quality of life. Subgroup analysis suggested that Fisher grade, and MMSE and MoCA scores at baseline in the subarachnoid hemorrhage patients and MMSE and MoCA scores at baseline in the ischemic patients affected quality of life in follow-up patients had achieved a good functional outcome (mRS 0-2). As a key result, we found that neurocognition and neurological function status at baseline were predictive factors of quality of life in intracranial VADA patients after endovascular reconstructive therapy.

It is well-known that the natural prognosis of intracranial VADAs is terrible, with a mortality rate of 23–35% during 5-year follow-up (19, 20). There is no doubt that early treatment of ruptured intracranial VADAs is strongly recommended due to the higher risk of recurrent hemorrhage and mortality (21, 22). However, as to unruptured intracranial VADAs, there is still debate as to whether invasive treatment is given to these patients. Previous investigations demonstrated that it is difficult to treat these unruptured intracranial VADAs once they had slowly progressed to hemorrhage or ischemia cases and that these patients will have a poor clinical outcome (23, 24). Thus, positive treatment for symptomatic intracranial VADA patients was advocated in our institution, particularly with regard to wide-necked and progressively growing aneurysms.

Both deconstructive and reconstructive measures can be achieved with novel endovascular interventions. Some researchers may favor the technique of trapping the parent vessel when the contralateral vertebral artery is codominant/dominant and is able to supply the posterior circulation independently. They consider that occlusion of blood supply to an aneurysm directly will decrease the rate of aneurysm recurrence and rebleeding (25, 26). Nevertheless, this approach is not without drawbacks. The first disadvantage is the possibility of coil migration, symptomatic infarction of posterior circulation, and rebleeding (27, 28). The second is that performing a balloon test occlusion (BTO) is often time-consuming and increases technical complexity, risk, and also the cost to patients (29). In fact, increasingly, neurosurgeons prefer to use a reconstructive strategy because of the low risk of mortality and morbidity. As we all know, therapy for small aneurysms involving a vertebral artery by using coils or stent–assisted coils remains a valuable option, achieving favorable results. In fact, some giant and complex VADAs have been treated with reconstructive endovascular therapy successfully, providing long-term benefit (30, 31). In this study, similar to the literature (32, 33), we acquired a satisfactory obliteration outcome and a low rate of postoperative complications and aneurysm recurrence. We also observed that there was a higher rate of partial obliteration in PICA-involving cases, which was in favor of the viewpoint that original PICA involvement is a risk factor for post-procedural recurrence (9). Therefore, we should perform a study to evaluate the association between recurrence and immediate obliteration grade in PICA-involving aneurysms in the future. Obviously, further studies consisting of larger patient cohorts and a longer follow-up period are required to confirm the results.

In line with previously published and reviewed data (34, 35), intracranial VADAs presenting with hemorrhagic or ischemic symptoms have an impact on functional, cognitive status and quality of life. Likewise, there are higher mRS scores for functional disability, corresponding well with lower MMSE and MoCA scores for neurocognition impairment at baseline in this study. Furthermore, mRS scores at baseline proved to be an independent factor for SS-QOL at follow-up, not only in our univariate analysis but also in the multivariate regression model. MMSE and MoCA most probably reflected lesion-associated cognitive impairments, in concordance with the literature (36) which described poststroke cognitive decline by global cognitive screening in MMSE and MoCA. Lower MMSE and MoCA scores were independent negative predictors of QOL at follow-up in the univariate analysis. In the multivariate analysis, only MMSE was a predictor for QOL, and a poor predictor, at that. These findings were in line with previous data on cognitive impairment in cerebellar hematoma, brain tumor, or cerebellar stroke patients (37, 38). The majority of patients obtained a good functional outcome at 6-month follow-up after surgery and showed a statistically significant difference in the mean value of mRS scores. There was a trend in the patients with elevated levels of MMSE and MoCA, though this was not significantly different.

One of the main findings was high prevalence of reduced QOL despite good functional outcome (mRS 0-2) in about 45.5% (*n* = 15) of group H and 51.4% (*n* = 19) of group I at follow-up. Because previous research has demonstrated no crucial role of neurovascular risk factors or sociodemographic factors in the QOL of VADA patients (39), we focused our attention on potential contributing factors such as neurological and neurocognitive variables. As the key result, our subgroup analysis of VADA patients at follow-up showed that a higher Fisher grade and lower MMSE and MoCA scores in group H, as well as lower MMSE and MoCA scores in group I, were associated with good functional outcome and bad QOL compared to those with good functional outcome and good QOL. In this study, we did not find any effect of vertebral artery stenosis or MRI-based infarction on the quality of life. However, there may be some factors that explain this consequence. First of all, besides the sample size being very small, it remains unclear how one can choose the best criteria for evaluating stenosis in a vessel, for example which method should be used to determine (1) the severity of stenosis (MRI and CT angiography or ultrasound) and (2) the length or site of stenosis or occlusion. Secondly, we simply assumed that brain infarction was likely to have an important role in quality of life. We used a very arbitrary method to evaluate the size of the infarction lesion. Apart from the limitations of the measurement methods, we assumed that the site of the infarcted area and the number of infarction lesions was much more important than the size.

Subarachnoid hemorrhage Fisher grade was observed as an important factor in our study, being significantly different in the bad QOL group in comparison with the good QOL group with good functional outcome in group H. As we all well-know, the severity of subarachnoid hemorrhage Fisher grade is associated with the rate of occurrence of delayed cerebral vasospasm. Some previous studies demonstrated that Fisher grade was a risk predictor for delayed cerebral infarction (40, 41). Therefore, we hypothesized that a higher Fisher grade might contribute to a higher possibility of delayed cerebral infarction, which would result in lower cerebral perfusion and would influence the rehabilitation of patients at follow-up. We can initiate further research to investigate the relationship of cerebral vasospasm after subarachnoid hemorrhage with quality of life in the future.

Importantly, our study also had several limitations. First of all, the statistical evidence is limited due to this being a single-center study and having a small sample size. Second, some deviation may exist in baseline data collection because patients were anxious and fearful in the acute stage at admission. Finally, additional potential factors relevant to outcome such as nutrition, economic situation, and family support, as well as social factors, were not taken into account in this study.

## Conclusion

In conclusion, endovascular reconstructive therapy is a safe and effective strategy for acute intracranial VADA patients, with a low rate of postoperative complications. Multiple factors may significantly impair QOL at 6-month follow-up. These lead to a reduced QOL in a significant percentage of patients despite good functional outcome. Our data suggest that neurocognition and neurological function status at baseline were of significant importance as predictive factors for quality of life in intracranial VADA patients after endovascular reconstructive therapy. Therefore, we should pay special attention to neuropsychiatric status after intracranial VADA patient treatment and enable timely therapeutic intervention to improve the quality of life.

## Data Availability Statement

All datasets generated for this study are included in the article/supplementary material.

## Ethics Statement

The studies involving human participants were reviewed and approved by Ethics Committee of the Anhui medical University. The patients/participants provided their written informed consent to participate in this study. Written informed consent was obtained from the individual(s) for the publication of any potentially identifiable images or data included in this article.

## Author Contributions

LG and BC performed the research and wrote the paper. YQ reviewed literature and selected studies based on inclusion and exclusion criteria. JL evaluated functional outcome, QOL, and radiology data. YHo evaluated cognitive status. YHu extracted and analyzed the data. HC and BC designed the study and revised the manuscript. All authors contributed to the article and approved the submitted version.

## Conflict of Interest

The authors declare that the research was conducted in the absence of any commercial or financial relationships that could be construed as a potential conflict of interest.
